# Dr. Waters' Statement of the Gardner Case

**Published:** 1880-02

**Authors:** George Franklin Waters

**Affiliations:** Missouri Dental Journal.


### ARTICLE V.

### *Dr. Waters' Statement Regarding his Treatment of Mr. George Arthur Gardner.*

Dr. Waters, of Boston, makes the following statement regarding his treatment in the case of Mr. George Arthur Gardner, of New York, whose recent death has attracted such wide-spread attention:

A great deal is being printed about the painful death of my kinsman and occasional patient, Mr. George Arthur Gardner, of New York. In such a case the investigations and discussions, instead of being conducted by irresponsible newspaper reporters, who publish, with more or less accuracy, the results of random interviews, should be made by the legal authorities, and with due formality. I beg permission to say this much, at this stage of the *quasi* investigation, in your columns:

Mr. Gardner came to my office on the 9th of September (I not having seen him for eight months previously,) suffering from neuralgic pains in the head and jaws, which he referred to a decayed tooth in the right inferior maxilla, that had been giving him trouble for three or four weeks. He said two dentists had made applications to the tooth at different times within three weeks, but without giving him much relief. Noting a soft, silky shimmer of the skin of his face I asked after his general health, and found that as good as usual, with the exception above noted. He seemed sure that the pulp of his tooth was in a high state of inflammation, and asked me to make an application for the purpose of allaying inflammation, before operating, as he feared the pain. On examination, I found *no* exposure of the pulp, nor any apparent opening into the pulp chamber from this cavity. But a dark circle around a gold filling in the crown looked suspicious, and, as it had a dark line extending forward to near the edge of this cavity, with a decided cloudiness bordering either side, I inferred the trouble to be located there. I found, upon removing the gold filling, a very extensive decay beneath and around it, penetrating the pulp chamber, which was filled with fine particles of food in a putrid condition. This was all carefully and thoroughly removed, and the discolored walls cut away so as to give free access to the pulp canals in the roots, in one of which I found a small portion of live pulp. The others were filled with a mass of fœtid matter, which was very carefully and thoroughly removed and the whole treated to a bath of bi-sulphate of soda, and that was followed by a very thorough washing with the saturated *aqueous* solution of carbolic acid. These materials were allowed ample time in which to do their work of disinfection and deodorization, and then the canals were filled with the soft, oily vaseline, and that protected by the form of oxy-chloride of zinc, called agate cement. When this had become sufficiently hard, the remainder, and by far the larger part, of the cavity was filled with Hill's stopping—a preparation of gutta percha. When I announced the completion of the filling Mr. Gardner seemed much surprised, and said that he had experienced no pain except when I was cleannig the canal, in which was a portion of live pulp, and that was of momentary duration. I was very careful not to wound the nutrient vessels of this small portion of humanity. Mr. Gardner was told that the entire operation was of a temporary nature, as the tooth was in an exceedingly precarious condition, and any operation while in this state, save that of extraction and replacement, would be of very doubtful duration. I also said that I wished to see the tooth again, in any event, before he went to New York that evening, that I might judge of the expediency of further treatment. In a few hours he came in again and said he thought I would have to loosen the filling a little, as he could not stand the pressure. I removed the gutta-percha, oxy-chloride of zinc, and washed out the vaseline. Then, fitting a piece of cork to the cavity, I made traction through it with a small syringe upon the contents of the dental canals. And, as it gave immediate relief, I decided that there was probably an oozing from the ruptured pulp vessels, and that it would consequently be improper to close the canals tightly, but that food and saliva must be kept out; so, after again washing with the saturated *aqueous* solution of carbolic acid, and applying a small quantity of nitrate of silver to the canals, I filled loosely the large cavity with Japanese bibulous paper, super saturated with vaseline, to the entire exclusion of air and moisture, yet not so as to prevent any gases or exudations that might develop in the roots from passing into the buccal cavity.

The same piece of cork was placed in the mouth of the cavity as a cover to the soft filling, and this condition was found by Dr. Marvin, as the papers have said, intact. If I had been in the habit of using arsenic, which I discarded 20 years since, and was entirely unscrupulous as to its use, and had the arsenic at hand, I should *not* have used it in such a case. I saw no indication that arsenic had been used at all. There was no sloughing of gums between the bicuspid and this molar, as there would doubtless have been if arsenic had been applied to this dishing mesial cavity, at the treatment which it received previous to his calling upon me. If there had appeared an opening into the pulp chamber from this cavity, I should have suspected the use of arsenic and removed the tooth for treatment as the only way of reaching the trouble. The indications of inflammation around the roots were not strongly marked when he called upon me, nor when he left, but yet sufficient to warn me of what might possibly occur, and accordingly I gave him positive instructions, if the pain returned to him, to have the traction treatment of the canals used again by the first dentist at hand, as by it the inflammatory symptoms seemed to be readily reached and controlled. Up to the time when Lewis was called in, on Saturday, there is nothing to show that Mr. Gardner was suffering from anything more serious than a severe and very troublesome alveolar abscess, the pointing of which Mr. Lewis says he saw and treated. Just what his treatment was we are left to infer by the subsequent results. A dentist would have opened the abscess and removed its contents, disinfecting and cleaning it to its source, even to the canals of the tooth. Every dentist must have occasion to treat cases very much worse than anything described by Mr. Lewis at his first meeting with this case. Just here we want more light, and these questions suggest themselves: What time Saturday did Lewis see the case? What was his treatment? Is he a medical student engaged in dissecting? Did he make a feeble attempt to open up that abscess? And if so, what with? An instrument used in dissecting? How long, in hours, was it from the time of viewing the case Saturday to the time in which he saw such dire results and condition on Sunday, when the pyæmia was marked and gangrene followed? Such a condition would be likely to result from the use of a dissecting knife that had not been properly cleaned, if used on tissues so engorged and clogged with effete matter.

I know of no other way in which such results could be obtained. I have seen and treated recently some bad cases of arsenical poisoning. The treatment is simplicity itself, as compared with that with which we have to combat the pyæmia of a dissecting wound. I am talking necessarily at random. Up to Saturday, the case was not, apparently, uncommon nor difficult to comprehend. What happened to him in the hands of this inexperienced medical student we do not know; we only know the sad results. For myself, I know, from more than thirty years of experience, that Mr. Gardner did not suffer harm from my treatment.

GEORGE FRANKLIN WATERS.

—*Missouri Dental Journal.*

